# Prevalence of diabetes mellitus and hypertension in people living with human immunodeficiency virus on antiretroviral therapy in Gweru district, Zimbabwe

**DOI:** 10.4102/phcfm.v12i1.2473

**Published:** 2020-08-11

**Authors:** Laston Gonah, Indres Moodley, Khumbulani Hlongwana

**Affiliations:** 1 Health Outcomes Research Unit, Discipline of Public Health Medicine, School of Nursing and Public Health, College of Health Sciences, University of KwaZulu-Natal, Durban, South Africa

**Keywords:** HIV, hypertension, diabetes mellitus, non-communicable diseases, prevalence, Zimbabwe

## Abstract

**Background:**

While antiretroviral therapy (ART) has markedly increased survival in people living with human immunodeficiency virus (PLHIV), emerging trends of co-existence of non-communicable diseases (NCDs) and HIV could negate the gains already achieved in controlling HIV.

**Aim:**

The study aimed to determine the prevalence of hypertension and diabetes mellitus in PLHIV on ART in Gweru district.

**Setting:**

Six high-volume ART sites in Gweru district under Midlands province in Zimbabwe.

**Methods:**

This was a cross-sectional study. Screening and data collection occurred over a 3-month cycle when all patients were expected to have visited the ART sites for their monthly ART drug supply. The process also allowed the identification of health system challenges regarding data management for HIV-NCD comorbidity. Poisson regression analysis was used to calculate NCD prevalence ratio (PR) in PLHIV.

**Results:**

Nearly 18 000 PLHIV registered for ART were identified. Hypertension (19.5%) and diabetes mellitus (8.4%) were the most common NCDs identified with a high proportion of those who did not know their diagnosis (over 50%). The prevalence of hypertension and/or diabetes mellitus among women was 74.9% compared to 25.1% in men (PR 3.22; 95% CI: 3.07–5.51, *p* = 0.0000). Other factors associated with increased prevalence of hypertension and/or diabetes mellitus were age group of ≥ 60 years (PR 2.5; 95% CI: 1.42–3.22, *p* = 0.00023), and duration of ≥ 5 years on ART (PR 6.4; 95% CI: 4.70–8.01, *p* = 0.0011). Separate data collection for NCDs and HIV was a key challenge affecting quantification of magnitude of HIV-NCDs comorbidity and subsequently management of NCDs in PLHIV.

**Conclusions:**

Indications of increasing prevalence of NCDs in PLHIV call for integrated electronic data management for HIV, TB and NCDs. This will allow active NCD case finding, and eventually improve prevalence data and treatment for HIV-NCD comorbidity. Future studies should focus on the health experiences and access to treatment in PLHIV diagnosed with NCDs; and to establish the accurate manner in which HIV status, ART and NCDs might be associated, through conducting a case control or cohort study.

## Introduction

Successful antiretroviral therapy (ART) has significantly contributed to viral suppression and markedly increased survival in people living with human immunodeficiency virus (PLHIV). Available evidence shows that because of increased investment for HIV/acquired immunodeficiency syndrome (AIDS) control, the proportion of PLHIV accessing ART worldwide increased significantly from 47% in 2010 to 62% in 2018, and a concomitant 33% decline in AIDS-related mortality.^1^ By the end of 2018, Zimbabwe witnessed an unprecedented increase in the proportion of PLHIV accessing ART (86.8% of 1.3 million PLHIV) with a corresponding 45% decrease in AIDS-related deaths since 2010.^1^ These statistics point to a positive trend of a general increase in survival among PLHIV largely because of ART.

While ART has markedly contributed to increased survival in PLHIV, emerging trends of co-existence of non-communicable diseases (NCDs) and HIV could negate the gains already achieved in controlling HIV. Current evidence suggests that PLHIV on ART are at increased risk of developing NCDs because of increased risk associated with longer survival, residual effect of ART drugs and from the effect of HIV itself.^2^ Epidemiological data on the magnitude of NCDs in PLHIV consistently point to higher prevalence rates of cardiovascular diseases (especially hypertension) and metabolic disorders (especially diabetes mellitus type 2) occurring in PLHIV.^3,4^ However, studies on the prevalence of NCDs in Africa are generally limited, and available figures on the prevalence of hypertension and diabetes mellitus in Zimbabwe were found to be largely inconsistent.

Zimbabwe has not yet implemented integrated management of HIV-NCD comorbidity. The gains already made in controlling HIV might be lost if the magnitude of other emerging and potentially life-threatening conditions like NCDs remain unknown. Against this background, the study sought to determine the prevalence of hypertension and diabetes mellitus, and how these are associated with age, sex and duration on ART among PLHIV in Gweru district.

## Methods

### Design and setting

This was a cross-sectional study conducted among PLHIV registered for ART in six high-volume ART sites which have the highest number of PLHIV collectively representing over 80% of all PLHIV who are on ART in Gweru district. Six sites (four urban and two rural) in Gweru district were purposively selected for the study. The four urban sites are administered by City Council Health Department, while the two rural sites are managed by the government of Zimbabwe Ministry of Health and Child Care.

### Study participants and recruitment

The study population consisted of all PLHIV aged 18 years and above, registered for ART at any of the six selected ART sites. Participants were recruited from the electronic patient monitoring system (ePMS) which captures patient’s demographic details (age, sex, physical address) together with HIV-related information. Those aged below 18 years were not considered for the study because of the complex consent process involved in enrolling minors, as well as because of the low risk for NCDs associated with younger age. All registered patients are expected to visit their respective ART site at least once in 3 months for their ART drug supply, thus presenting the opportunity for data collection.

### Data collection

**FIGURE 1:**
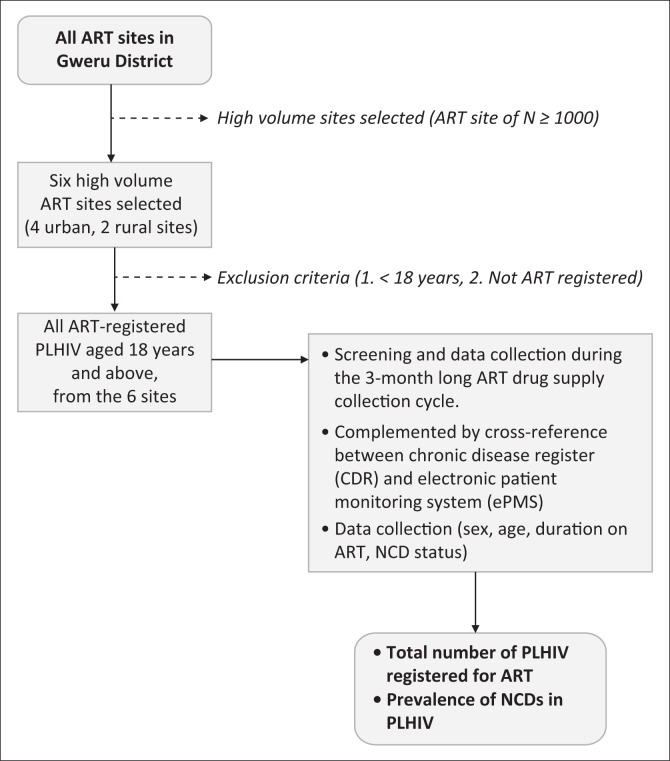
Flowchart: Participant selection and data collection.

Screening and data collection occurred over a 3-month period (January 2019 to March 2019) during which all patients were expected to have visited the ART sites for their monthly ART drug supply (see Figure 1).

To identify patients with HIV-NCD comorbidity, patients’ booklets, where NCD diagnoses such as hypertension, diabetes mellitus, cancers and mental conditions are recorded at each visit, were reviewed. Self-reported NCD cases were verified by requesting the patient to provide supporting evidence. A healthcare worker responsible for patient consultation was responsible for data collection which was recorded by using patients’ unique ART numbers for identification, without interrupting normal daily activities. During the consultation, patients were also screened for hypertension and diabetes mellitus, where positive results were followed up to confirm diagnosis.

Existing data were complemented by undertaking cross-reference between the chronic disease register (CDR), which is hard-copy-based, and the ePMS. The CDR only captures data for all other non-AIDS-related NCDs and not HIV data. Duplicates identified from the two processes were removed. Efforts were made to contact patients not reporting for their ART drug supply within the 3-month visit period, and untraceable cases, transfers or loss to follow-ups were noted.

### Data analyses

Data capturing and descriptive data analysis were performed in SPSS version 13 to determine proportions and frequencies. Data were summarised in tables and graphs as appropriate. Poisson regression analysis was performed to calculate NCD prevalence ratio (PR) by sex, age, and duration on ART among PLHIV.

### Ethical consideration

Ethics approval for the study was obtained from the Biomedical Research Ethics Committee of the University of KwaZulu-Natal (Reference: BE086/19) and Ministry of Health and Child Care Head office, and participation in the study was on a voluntary basis. Written informed consent was sought from potential participants, and confidentiality was maintained throughout the study by removal of personal identifiers after entry into the electronic database and use of non-identifiable coded numbers. Further, all data were password protected in the storage of the electronic participant database. All patients identified with unmet health needs were referred for appropriate care.

## Results

### Participants’ characteristics and descriptive analysis

A total of 18 233 PLHIV were registered in the six study ART sites. The number of patients per site ranged from 710 to 5871, as shown in Table 1. Of the total number of registered HIV patients, 97.5% (17 784) were receiving ART at the time of the study. Among the reasons for those patients who were not receiving ART were those with presumptive tuberculosis (TB) awaiting TB investigation results before ART initiation; those who opt out of ART with ongoing counselling; and those who were tested before the test-and-treat policy criterion. In the latter group of patients, some could not be traced or may have moved to other sites. Therefore, the data analysis and statistics were based on the 17 784 PLHIV on ART.

**TABLE 1:** Participant characteristics and descriptive statistics.

Variable	ART centre											
	Urban 1†		Urban 2		Urban 3		Urban 4		Rural 1		Rural 2	
	*n*	%	*n*	%	*n*	%	*n*	%	*n*	%	*n*	%
**Total number of PLHIV % of PLHIV not on ART**	710	0.42	5871	1.04	5025	5.60	3915	0.90	1214	2.60	1498	2.70
**Total number of PLHIV registered on ART**	707	-	5810	-	4745	-	3880	-	1183	-	1459	-
Female	460	65.1	3619	62.3	2722	57.4	2533	65.3	752	63.6	949	65.0
Male	247	34.9	2191	37.7	2023	42.6	1347	34.7	431	36.4	510	35.0
**ART-registered PLHIV and hypertension**	143	20.2	1178	20.3	917	19.3	740	19.1	223	18.9	267	18.3
Female	102	71.3	826	70.1	681	74.3	547	73.9	175	78.5	215	80.5
Male	41	28.7	352	29.9	236	25.7	193	26.1	48	21.5	52	19.5
**ART-registered PLHIV and diabetes**	61	8.6	529	9.1	417	8.8	316	8.2	77	6.5	94	6.4
Female	48	78.7	397	75.0	324	77.7	226	71.5	66	85.7	80	85.1
Male	13	21.3	132	25.0	93	22.3	90	28.5	11	14.3	14	14.9
**ART-registered PLHIV & hypertension & diabetes**	46	6.5	326	5.6	316	6.7	224	5.8	56	4.7	75	5.1
Female	34	73.9	246	75.5	232	73.4	148	66.1	38	67.9	54	72.0
Male	12	26.1	80	24.5	84	26.6	76	33.9	18	32.1	21	28.0
**ART-registered PLHIV and other NCDs (asthma, psychiatric conditions, epilepsy)**	10	1.4	41	0.70	31	0.65	24	0.62	8	0.68	11	0.75
Female	7	70.0	30	73.2	23	74.2	19	79.2	6	75.0	8	72.7
Male	3	30.0	11	26.8	8	25.8	5	20.8	2	25.0	3	27.3

† , selected even though *n* < 1000 PLHIV because it was recently established, and hence, the total number of PLHIV at that site was considered high.

The study population was composed of mostly urban (85.14%) patients, and the majority of PLHIV on ART were women (62.10%). Participants’ age was categorised into five age groups: 18–39, 40–49, 50–59 and ≥ 60 years. The 18–39 years age category represented the majority (52.25%) of the PLHIV, followed by the 40–49 years age group (30.1%) (Table 2).

**TABLE 2:** Demographic data of people living with human immunodeficiency virus included in the study.

Variable	*n*	%
**Sex**		
Female	11 035	62.10
Male	6749	37.9
**PLHIV on ART by age category (years)**		
< 39	9293	52.25
40–49	5352	30.10
50–59	2294	12.90
≥ 60	845	4.75
**Place of residence**		
Urban	15 142	85.14
Rural	2642	14.86
**NCD outcome**		
Hypertension	2670	15.0
Diabetes mellitus	374	2.1
Diabetes mellitus and hypertension	75	0.42
Other NCDs	125	0.70
**Duration on ART**		
< 1 year	1156	6.5
1–5 years	5211	29.3
> 5 years	11 417	64.2

### Prevalence of non-communicable diseases in people living with human immunodeficiency virus

Non-communicable disease categories assessed were mainly hypertension, diabetes and a few others. Overall, the proportion of PLHIV receiving ART diagnosed with hypertension was 19.5% (3468 cases out of 17 784), where the majority (73.4%) of these patients were women compared to men (26.6%). The prevalence of hypertension in urban ART sites was 19.7% and 18.5% in rural sites. Overall, the proportion of undiagnosed hypertension was 64.3% (i.e. 64.3% of the 3468 hypertensive cases did not know their status before screening).

Diabetes mellitus was found in 1494 out of 17 784 PLHIV (8.4%), the majority of them being female patients (76.4%). The prevalence of diabetes mellitus in PLHIV on ART in urban sites was 8.7% and 6.5% in rural ART sites.

The proportion of PLHIV receiving ART with a confirmed diagnosis for both hypertension and diabetes mellitus was 5.9%. The majority of comorbid hypertension and diabetes mellitus cases were female patients (72.1%). The prevalence of comorbid diabetes mellitus and hypertension in urban ART sites was 6.0%, and it was 5.0% in rural ART sites.

With regard to other NCDs in PLHIV, some cases of asthma, mental health conditions and epilepsy contributed 0.7% of PLHIV on ART (Tables 1 and 2). The majority of the cases with other NCDs were female patients (74.4%) compared to male patients (25.6%).

Chi-square results for the relationship between NCD status and age, sex, duration on ART and geographical location are shown in Table 3.

**TABLE 3:** Association of non-communicable disease status by age, sex and duration on antiretroviral therapy.

Variable	NCD present (hypertension and/or diabetes mellitus)				*p*-value (*α* = 0.05)
	Yes		No		
	*n*	%	*n*	%	
Patients	3919	22.0	13 865	78.4	-
**Sex**					0.0000
Male	984	25.1	5765	41.6	-
Female	2935	74.9	8100	58.4	-
**Age (years)**					0.00001
< 39	586	15.0	8707	62.8	-
40–49	1927	49.2	3425	24.7	-
50–59	785	20.0	1509	10.9	-
≥ 60	621	15.8	224	1.6	-
**Area of residence**					0.0079
Urban	3389	86.5	11 753	84.8	-
Rural	530	13.5	2112	15.2	-
**Duration on ART (years)**					0.00001
< 1	116	3.0	1040	7.5	-
1–5	834	21.3	4377	31.6	-
> 5	2969	75.7	8448	60.9	-

#### Non-communicable disease prevalence (hypertension and/or diabetes) by age, sex and duration on antiretroviral therapy

Poisson regression analysis showed that the prevalence of hypertension and/or diabetes mellitus among female patients was 74.9% compared to 25.1% in male patients (PR 3.22; 95% CI: 3.07–5.51, *p* = 0.0000). Other factors associated with increased prevalence of hypertension and/or diabetes mellitus were age group of ≥ 60 years (PR 2.5; 95%CI: 1.42–3.22, *p* = 0.00023) and duration of ≥ 5 years on ART (PR 6.4; 95% CI: 4.70–8.01, *p* = 0.0011).

### Health system factors affecting the observed prevalence of non-communicable diseases

It was found that patients’ HIV-related data and NCD-related data were being collected and stored separately in different databases at all ART study centres. HIV- and ART-related data for PLHIV are manually captured in patient Green Books and electronically captured in the ePMS. However, data for all NCDs at health facilities are manually captured in the CDR (hard-copy-based) regardless of HIV status. This separate data management system does not allow for easy identification and quantification of the magnitude of NCDs in PLHIV, and it is not possible to easily identify anyone with an NCD who is HIV positive, because the ePMS and CDR register do not allow the simultaneous capturing of patient’s NCD and HIV status.

There was evidence of some patients with HIV-NCD comorbidity being registered for NCD care at a different health facility to where they receive their ART care. A significant proportion (1017 out of 3468 PLHIV, representing 29.3%) of people with HIV diagnosed with hypertension and/or diabetes mellitus were not registered for hypertension or diabetes care at the same health centre where they get ART medication. This came out during cross-referencing of self-reported NCD diagnoses (supported by relevant medical proof), the ePMS and the CDR.

## Discussion

The study sought to determine the prevalence of hypertension and diabetes mellitus in PLHIV who were registered for ART and the association with age, sex and duration on ART. Health system factors affecting identification and quantification of NCDs in PLHIV at healthcare facility level were also assessed.

In this study, the prevalence of hypertension in PLHIV on ART was 19.5%. This rate is lower compared to findings from the majority of studies conducted outside Africa, in countries such as Asia, United States and Europe, where prevalence rates of hypertension of up to 60% in PLHIV were found.^5,6,7,8^ Other studies conducted in a similar Zimbabwean context showed marked variations in the prevalence rates of hypertension in PLHIV, ranging from 10% to 30%.^9,10,11^ In this study, the prevalence of diabetes mellitus in PLHIV was 8.4%. Similar lower trends were observed for hypertension. Evidence shows higher prevalence rates of diabetes mellitus in PLHIV of up to 28% in the studies conducted in Asia, Europe and the UnitedStates.^6,7,12,13^ In contrast to studies conducted within Zimbabwe, the prevalence rates for diabetes mellitus ranged from 1% to 8%.^9,11,14^ In general in sub-Saharan Africa, where the majority of PLHIV live, lower rates of NCDs were observed.^1^

In this study, a significant proportion of hypertension did not know their diagnosis, as supported by World Health Organization.^4^ Higher prevalence rates observed for NCDs in PLHIV outside Africa, in high-income countries, might be because of improved screening and diagnostic services leading to identification of more cases, and better disease management resulting in improved survival of PLHIV. Although the exact reasons are not yet known, the lower and inconsistent rates observed for Zimbabwe might be explained in part by the possibility of limited capacity to screen and diagnose NCDs in PLHIV because of the economic crisis faced by the country.^15^ Observed prevalence of NCDs is influenced by a variety of factors. High mortality rates because of poor disease management, together with limited capacity to screen and diagnose NCDs (because of lack of appropriate human, financial and material resources), have the potential to result in lower prevalence rates being observed.^16^ On the other hand, better NCD management, which is also associated with greater capacity to screen and diagnose NCDs, can result in increased survival leading to higher prevalence rates being observed in high income countries (HICs).^4^ Further studies are required to understand the reasons for higher prevalence rates of NCDs in PLHIV in HICs compared to low-income countries, and the capacity of ART centres in Zimbabwe to screen and diagnose NCDs in PLHIV.

This study did not compare prevalence of diabetes mellitus and hypertension in PLHIV to HIV negative people. However, other studies conducted in Zimbabwe show lower prevalence of hypertension and diabetes mellitus in the general population compared to PLHIV.^11^ The higher prevalence of NCDs in PLHIV can be explained in part by the age-related degenerative changes accelerated by HIV, progressive immune dysfunction, accumulation of ART drug toxins, effects of ongoing inflammations as well as long-term viral infection associated with PLHIV.^2^ This study found statistically significant associations between older age and longer duration on ART as significant factors independently associated with increased prevalence for hypertension and diabetes mellitus. More studies are recommended to assess how ART regimen type may influence differences in NCD prevalence rates between those with longer duration compared with those with shorter duration on ART. Future studies need to determine and compare prevalence of NCDs in PLHIV with that for HIV-negative people, so as to assess the risk of development of NCDs in PLHIV. It might also be worthwhile to compare healthcare costs for PLHIV-NCD comorbidity (as cases) with healthcare costs for PLHIV alone (as controls), to explain the effect of NCDs on healthcare costs in PLHIV.

Some cases of other NCDs, mainly asthma, epilepsy and mental health disorders, were observed in PLHIV during the study. However, the combined magnitude of these conditions in PLHIV was found to be very low. More research evidence is required on the capacity of ART centres to screen and diagnose these NCDs both in the general population and in PLHIV to substantiate these findings.

Significant gender differences were noted in the study, where the majority (more than 60%) of registered patients on ART from both rural and urban ART sites were women. Generally, the proportion of adult women is known to be higher in the general population.^17^ Evidence also shows that women are at an increased risk of HIV infection compared to men.^1,18,19^ Again, the male or female differences in the prevalence of both hypertension and diabetes mellitus may have been influenced in part by the fact that women are more likely to be overweight or obese compared to men.^18^ Overweight or obesity is a well-known risk factor for development of both hypertension and diabetes mellitus.^4^ Besides the mentioned justification factors for the lower proportions of men observed in the study, male gender has been shown to be associated with poor healthcare-seeking behaviours.^20^ Men may participate less in HIV and NCD screening and treatment programmes, resulting in lower than actual proportions being identified. Context-specific research evidence is required to explain differences in demographic characteristics and healthcare-seeking behaviours between men and women in Zimbabwe. Such studies will also come up with reasons for gender differences in observed prevalence rates for NCDs in PLHIV in the Zimbabwean context.

Key healthcare system factors affecting identification and quantification of NCDs at health facility level were separate data collection and management for HIV and NCDs, and instances where the same patient with comorbid HIV and NCD(s) could be registered for ART at one facility while registered for NCD(s) care at another health centre. The databases of PLHIV were being managed separately from the database for NCDs, making it difficult to easily identify HIV-positive people with NCDs. This situation makes comprehensive patient monitoring difficult. Successful HIV/AIDs control relies on comprehensive management of the patient, which include uninterrupted access to and adherence to ART, and screening and treatment of comorbid conditions in an integrated manner.^21,22^ Studies conducted elsewhere that assessed the feasibility and cost-effectiveness of integrated HIV and NCD care found it to be worthwhile.^23,24,25,26^

Studies assessing the feasibility and cost effectiveness of integrating NCD treatment into existing HIV care programmes are recommended for Zimbabwe. Further studies are required that will assess the feasibility of integrating the ePMS and the CDR into one database, in order to improve data availability and quality for comorbid HIV and NCDs in Zimbabwe. Again, factors contributing to PLHIV registering for ART at a different health facility than where they register for NCDs and other health conditions need to be explored in detail.

### Study limitations

The study lacked a comparison group of HIV-negative people to compare prevalence of NCDs in PLHIV and in HIV-negative people. Future study will compare healthcare costs in people living with comorbid HIV and NCDs with that for HIV-positive people without NCDs. Studies aimed at assessing the quality of primary care and the improvement of quality of life for PLHIV-NCD comorbidity are recommended.

Other important factors in explaining the occurrence of NCDs like individual patients’ Body Mass Index, smoking status, socio-economic status and diet, among others, were not explored, because of the large size of the study population negatively affecting feasibility of collecting data for these variables. These will be explored in detail in subsequent findings from another study.

## Conclusion

The indications of increasing prevalence of NCDs in PLHIV call for an integrated electronic data management for HIV, TB and NCDs. This will allow active NCD case finding and eventually improve prevalence data and treatment for HIV-NCD comorbidity.
